# Overexpression of Mitochondrial Calcium Uniporter Causes Neuronal Death

**DOI:** 10.1155/2019/1681254

**Published:** 2019-10-16

**Authors:** Veronica Granatiero, Marco Pacifici, Anna Raffaello, Diego De Stefani, Rosario Rizzuto

**Affiliations:** Department of Biomedical Sciences, University of Padova, Via Ugo Bassi 58B, Padova, Italy

## Abstract

Neurodegenerative diseases are a large and heterogeneous group of disorders characterized by selective and progressive death of specific neuronal subtypes. In most of the cases, the pathophysiology is still poorly understood, although a number of hypotheses have been proposed. Among these, dysregulation of Ca^2+^ homeostasis and mitochondrial dysfunction represent two broadly recognized early events associated with neurodegeneration. However, a direct link between these two hypotheses can be drawn. Mitochondria actively participate to global Ca^2+^ signaling, and increases of [Ca^2+^] inside organelle matrix are known to sustain energy production to modulate apoptosis and remodel cytosolic Ca^2+^ waves. Most importantly, while mitochondrial Ca^2+^ overload has been proposed as the no-return signal, triggering apoptotic or necrotic neuronal death, until now direct evidences supporting this hypothesis, especially *in vivo*, are limited. Here, we took advantage of the identification of the mitochondrial Ca^2+^ uniporter (MCU) and tested whether mitochondrial Ca^2+^ signaling controls neuronal cell fate. We overexpressed MCU both *in vitro*, in mouse primary cortical neurons, and *in vivo*, through stereotaxic injection of MCU-coding adenoviral particles in the brain cortex. We first measured mitochondrial Ca^2+^ uptake using quantitative genetically encoded Ca^2+^ probes, and we observed that the overexpression of MCU causes a dramatic increase of mitochondrial Ca^2+^ uptake both at resting and after membrane depolarization. MCU-mediated mitochondrial Ca^2+^ overload causes alteration of organelle morphology and dysregulation of global Ca^2+^ homeostasis. Most importantly, MCU overexpression *in vivo* is sufficient to trigger gliosis and neuronal loss. Overall, we demonstrated that mitochondrial Ca^2+^ overload is *per se* sufficient to cause neuronal cell death both *in vitro* and *in vivo*, thus highlighting a potential key step in neurodegeneration.

## 1. Introduction

Ca^2+^ is the key intracellular messenger that regulates neuronal functions. Changes in cytosolic Ca^2+^ concentration ([Ca^2+^]) are controlled by either influx from the extracellular space (triggered by the opening of voltage- or ligand-gated Ca^2+^ selective channels) or release from the intracellular store such as the endoplasmic reticulum (through the IP_3_ or ryanodine receptors). The spatiotemporal patterns of cytosolic Ca^2+^ signals are strongly shaped by both proteins that bind and transport Ca^2+^ as well as by intracellular organelles, especially mitochondria. Thanks to their highly negative membrane potential, mitochondria can rapidly accumulate large amount of Ca^2+^ through a highly selective, ruthenium red-sensitive channel named mitochondrial Ca^2+^ uniporter (MCU) [1–3]. Accordingly, mitochondria can act as Ca^2+^ sponges and buffer either local or global increases in cytosolic [Ca^2+^]. Inside the organelle matrix, Ca^2+^ plays a pleiotropic role, since mitochondrial Ca^2+^ is a priming signal that can control many organelle features, including morphology, energy supply, ROS production, and cell death [4, 5]. Although only in few familial diseases the affected genes encode for proteins directly involved in Ca^2+^ homeostasis, there are many pathological conditions in which the defect is not primarily concerned with Ca^2+^ handling but their phenotypic manifestations are thought to depend on alterations of cellular [6] or mitochondrial [7] Ca^2+^ homeostasis. In this scenario, the situation of the neurodegenerative diseases is particularly telling and challenging at the same time. In most of the cases, they are sporadic disorders of unknown cause, although a subset of cases are genetically inheirited, thus representing an ideal model to study disease mechanism, at least in principle. However, genetic forms of Alzheimer's and Parkinson's diseases (AD and PD, respectively) are due to mutations in a variety of conceptually distant molecular elements (proteases, such as the presenilins; kinases, such as PINK1 and LRRK2; ubiquitin-ligases, such as Parkin; redox-sensitive chaperones, such as DJ-1), all converging in mitochondrial dysfunction as the cellular trigger of neuronal cell death. In this complex scenario, further complicated by a highly debated literature, a link can be traced with mitochondrial Ca^2+^ overload as a potential common pathway of the pathogenic route. In the case of familial forms of AD, some pathogenic mutations have been clearly linked to the impairment of cellular Ca^2+^ homeostasis [8, 9]. While in the last decades, a general consensus emerged supporting the hypothesis that an excessive Ca^2+^ accumulation into mitochondrial matrix, probably concomitantly with other toxic insults, may initiate the release of proapoptotic factors from mitochondria that eventually lead to cell death [5]. Until now direct evidences supporting mitochondrial Ca^2+^ overload as a trigger of neuronal cell death *in vivo* are still lacking. Only few studies have addressed the role of MCU in neurons. Hardingham and coworkers convincingly showed that MCU levels nicely correlate with NMDA sensitivity in primary hippocampal neurons [10] and that the transcription of MCU complex components is controlled by neuronal activity [11]. Similar results have also been obtained in cerebellar granule neurons exposed to oxidative stress [12]. Here, we investigated the specific role of mitochondrial Ca^2+^ overload, induced by MCU overexpression, in the neuronal degeneration. We show that neurons are extremely sensitive to mitochondrial Ca^2+^ overload-mediated cell death both *in vitro* and *in vivo*. Overall, our data suggest that exagerated mitochondrial Ca^2+^ uptake plays *per se* a pivotal role in priming neurodegeneration.

## 2. Materials and Methods

### 2.1. Culture and Transfection of Mouse Primary Cortical Neurons

All experiments were performed on primary cortical neurons from p0-p2 newborn C57Bl/6J mice. Briefly, the brain cortex of newborn mice were isolated using a stereomicroscope and digested with trypsin at 37°C. After two digestion steps, cells were counted and seeded on poly-L-lysine-coated glass coverslips. Neurons were cultured in MEM (Thermo Fisher Scientific), supplemented with 10% horse serum (Thermo Fisher Scientific), N2 supplement (Thermo Fisher Scientific), B27 supplement (Thermo Fisher Scientific), sodium pyruvate (Thermo Fisher Scientific), biotin, glucose, L-glutamine, penicillin, and streptomycin for 5 DIV before transfection or infection. Neurons were transfected with Lipofectamine 2000 (Thermo Fisher Scientific), according to the manufacturer's instruction. Experiments were carried out 24 hours after transfection. All chemicals were purchased from Sigma-Aldrich, unless otherwise specified. The pcDNA3.1-MCU-flag expression construct was described in [2].

### 2.2. Ca^2+^ Imaging

In depolarization-induced Ca^2+^ dynamic measurements, mouse primary cortical neurons at 5 DIV were cotransfected with 4mtD1cpV as probe [13], and with either empty pcDNA3.1 vector as control or pcDNA3.1-MCU-flag for the MCU overexpression. 24 hours after transfection, primary neurons were mounted in an open-bath custom-made imaging chamber and maintained in KRB (in mM: 135 NaCl, 5 KCl, 1 MgSO_4_, 0.4 K_2_HPO_4_, 20 HEPES, 1 CaCl_2_, pH = 7.4). Cells were then stimulated by perfusing an isosmotic-modified KRB containing 50 mM KCl (NaCl concentration was decreased accordingly). At the end of each experiment, ionomycin (5 *μ*M) (dissolved in a Ca^2+^-free KRB containing 500 *μ*M EGTA) was first applied to completely discharge the stores (*R*_min_). Then, a saturating CaCl_2_ concentration (5 mM) is added (*R*_max_), in order to verify the dynamic range of the probe. Analysis was performed with the Fiji distribution of ImageJ [14]. CpVenus and CFP images were subtracted of background signals and distinctly analyzed after selecting proper regions of interest (ROIs) on each cell (identified based on their morphology). The ratio between cpVenus and CFP emission was calculated (*R*). Alternatively, data are shown as normalized fold change of the ratio (Δ*R*/*R*_0_), where *R*_0_ is the ratio at the beginning of the measurement. Data and sample size are provided in Table 1. Cells expressing the fluorescent probes were analyzed using an inverted Zeiss Axiovert 100 TV equipped with a 40x/1.3N.A. Plan-NeoFluar objective. The probe was excited by a LED-based illumination device (OptoLED, Cairn Research) with a 436/20 nm bandpass filter. Donor and acceptor wavelengths were separated by a beamsplitter device (Optosplit II, Cairn Research) using a 480/40 nm filter for the CFP, a D505 dichroic mirror, and a 535/30 nm filter for the cpVenus. Images were collected with a front-illuminated CCD camera (Photometrics CoolSnap ES2). The system was controlled by MetaFluor 6.3 software (Universal Imaging) and assembled by Crisel Instruments. Exposure time and frequency of image capture varied from 100 ms to 300 ms and from 0.5 to 1 Hz, respectively, depending on the intensity of the fluorescent signal and on the desired temporal resolution.

In basal [Ca^2+^] measurements, mouse primary cortical neurons at 5 DIV were cotransfected with either GCamp6f or 2mtGCamp6m probes [15, 16], together with either empty vector mCherry or MCU-mCherry for the MCU overexpression. Resting cytosolic Ca^2+^ was measured in absence or in presence of Ca_v_1 (nimodipine 5 *μ*M, TOCRIS) or Ca_v_2 inhibitors (GVIA 2 *μ*M and M7C 1 *μ*M for Ca_v_2.2 and Ca_v_2.1, respectively, Bachem). 24 hours after transfection, primary neurons were mounted in an open-bath custom-made imaging chamber and maintained in KRB. Imaging was performed on a Zeiss Axiovert 200 microscope equipped with a 40×/1.3N.A. Plan-NeoFluar objective. Excitation was performed with a DeltaRAM V high-speed monochromator (Photon Technology International) equipped with a 75 W xenon arc lamp. Images were captured with a high-sensitivity Evolve 512 Delta EMCCD (Photometrics). The system is controlled by MetaMorph 7.5 (Molecular Devices) and was assembled by Crisel Instruments. Mouse primary cortical neurons were alternatively illuminated at 474 and 410 nm, and fluorescence was collected through a 515/30 nm bandpass filter (Semrock). Exposure time was set to 200 ms at 474 nm and to 400 ms at 410 nm, in order to account for the low quantum yield at the latter wavelength. At least ten fields were collected per coverslip, and each field was acquired for 10 s (1 frame/s). Analysis was performed with the Fiji distribution of ImageJ [14]. Both images were background corrected frame by frame by subtracting mean pixel values of a cell-free region of interest. Data are presented as the mean of the averaged ratio of all time points, as described previously [17]. Data and sample size are provided in Table 1.

For Fura-FF measurements, mouse primary cortical neurons at 5 DIV were infected with synapsin-driven pEGFP (synGFP) or MCU-GFP (synMCU-GFP) adenoviral particles. After 48 hours, primary neurons were loaded with a 5 *μ*M Fura-FF-AM in KRB for 30 min. After washing, cells were illuminated alternately at 340 and 380 nm, and fluorescence was monitored at 510 nm. Imaging was performed on an Olympus IX71/IX5 inverted microscope equipped with a xenon light source (150 W) for epifluorescence illumination. Images were collected with a digital camera with an exposure time of 200 ms using a 40x/1.3N.A. oil immersion objective (Olympus). Data were acquired and analyzed using CellR software (Olympus). Sequential digital images were acquired every second for 3 minutes (in order to well visualize the rapid Ca^2+^ uptake peak after glutamate treatment) and then every 3 minutes till the end of the experiment. Analysis was performed with the Fiji distribution of ImageJ [14]. Both images were background corrected frame by frame by subtracting mean pixel values of a cell-free region of interest. Data are presented as the mean of the averaged ratio ± S.E. of all time points. Data and sample size are provided in Table 1.

### 2.3. Immunofluorescence

After 5 DIV, mouse primary cortical neurons were transfected with mtRFP as mitochondrial marker and with either empty pcDNA3.1 vector as control or pcDNA3.1-MCU-flag for the MCU overexpression. 24 hours after transfection, primary neurons were treated or not, as indicated, with glutamate (100 *μ*M) for one hour and then fixed with 4% formaldehyde solution, permeabilized, and blocked in PBS containing 1% BSA, 2% goat serum, and 0.3% Triton X-100. Cells were then incubated with primary antibody anti-flag (1 : 200, Sigma-Aldrich), overnight at 4°C. The appropriate isotype-matched Alexa Fluor conjugated secondary antibodies (Thermo Fisher Scientific) were used, and coverslips were mounted with ProLong Gold Antifade reagent (Thermo Fisher Scientific).

Free-floating mouse brain slices (60 *μ*m thick) were permeabilized and blocked in PBS containing 1% BSA, 10% goat serum, and 0.3% Triton X-100. Then, slices were incubated with primary antibodies anti-GFP (1 : 200, Sigma-Aldrich), anti-MAP2 (1 : 100, Abcam), anti-CD45 (1 : 100, Abcam), and anti-GFAP (1 : 100, Dako) overnight at 4°C in free-floating and washed 3 times with PBS. The appropriate isotype-matched Alexa Fluor conjugated secondary antibodies (Thermo Fisher Scientific) were used, and coverslips were mounted with Prolong (Thermo Fisher Scientific). Analysis was performed with the Fiji distribution of ImageJ [14]. Quantification was made by measuring signal intensity above a certain threshold (30 for TUNEL, 20 for CD45, and 30 for GFAP) and dividing it by GFP signal intensity. Data and sample size are provided in Table 1. Data are expressed as mean ± S.E.

### 2.4. Adenovirus Production

Adenoviral particles were developed using the AdEasy Adenoviral vector system [18]. The vector pShuttle (Agilent #240006) was engineered to express either an enhanced green fluorescent protein or a MCU-EGFP chimera under the control of the human *SYNAPSIN* promoter (synGFP and synMCU-GFP, respectively). The resulted plasmid was linearized by digestion with Pme I, and subsequently co-transformed into *E. coli* BJ5183 cells together with the adenoviral backbone plasmid pAdEasy-1. Recombinants were selected for kanamycin resistance, and recombination is confirmed by restriction analysis. Purified recombinant Ad plasmid DNA was digested with PacI to expose its inverted terminal repeats (ITR) and then used to transfect adenovirus packaging cell line (293 HEK cells). Recombinant adenoviruses were typically generated within 7 to 12 days. Transfected cells were collected, lysed through freeze-thaw cycles, and centrifuged to remove cellular debris. The supernatant was then used for large-scale virus preparation.

For the stereotaxic injection, the same viruses were further purified through CsCl gradient centrifugation. The final viral titers were 6.32 × 10^10^ PFU/ml and 5.76 × 10^10^ PFU/ml for synGFP and synMCU-GFP, respectively. For each mouse, 0.85 *μ*l and 1 *μ*l of each preparation were injected.

### 2.5. Apoptotic Counts

After 5 DIV, mouse primary cortical neurons were infected with synGFP or synMCU-GFP adenoviral particles. After 48, 72, and 96 hours, cells were fixed with 4% formaldehyde solution and then mounted with ProLong Gold Antifade reagent (Thermo Fisher Scientific). For each coverslip, confocal images (Leica SP5-TCS-II) of twenty random fields were acquired. We then counted the number of GFP-positive neurons and calculated the percentage of GFP-positive neurons normalized on the first time point (48 hours). Data and sample size are provided in Table 1.

### 2.6. TUNEL Assay

After 5 DIV, mouse primary cortical neurons were infected with synGFP or synMCU-GFP adenoviral particles. 48, 72, and 96 hours after the infection, cells were treated or not, as indicated, with glutamate (100 *μ*M) for one hour and then fixed with 4% formaldehyde solution followed by permeabilization with 0.25% Triton X-100. Staining was performed with TUNEL assay kit (Thermo Fisher Scientific) according to the manufacturer's protocol. For each coverslip, confocal images (Leica SP5-TCS-II) of twenty random fields were acquired. We then calculated the percentage of TUNEL-positive neurons over GFP-positive cells (mean ± S.E.). Data and sample size are provided in Table 1.

### 2.7. Measurements of Mitochondrial *ΔΨ*

After 5 DIV, mouse primary cortical neurons were infected with synGFP or synMCU-GFP adenoviral particles. 48 hours after infection, primary neurons were loaded with TMRM probe (20 nM) for 30 minutes at 37°C. Images were taken on an inverted microscope (Zeiss Axiovert 200) equipped with a Plan-NeoFluar 40x/1.3N.A. objective, a Photometrics Evolve Delta EMCCD, and a 75W Xenon arc lamp coupled to a monocromator (PTI Deltaram V). The system was assembled by Crisel Instruments (Rome, Italy). TMRM excitation was performed at 560 nm, and emission was collected through a 590-650 nm bandpass filter. Images were taken every 5 seconds with a fixed 200 ms exposure time, for about 10 minutes after excitotoxic stimulus (glutamate 100 *μ*M). At the end of each experiment, CCCP (10 *μ*M) was added to dissipate mitochondrial membrane potential. Data are expressed as half-time of the decay in TMRM fluorescence (mean ± S.E.). In the case of TMRM data provided (acquired 48 hours after infection), we analyzed TMRM signal only from cells with intact membrane potential. Dead cells did not contribute to the overall signal in the analysis because (i) they detached from the coverslip after washes and/or (ii) they did not load with TMRM. Data and sample size are provided in Table 1.

### 2.8. Stereotaxic Injection

Stereotaxic injections were performed as previously described [19]. Briefly, thin plastic tubing is attached to the top of the glass micropipette in the holder and a syringe attached to the other end of the plastic tubing is used for virus aspiration or injection. Young adult C57Bl/6J mice (1 month of age) were anesthetized by intraperitoneal injection of tiletamine hydrochloride+zolazepam hydrochloride (Xilor) (20-40 mg/kg) and xylazine (Zoletil) (0.5 mg/kg) and fixed with ear bars. Under a dissecting microscope, an incision was performed to expose the skull. Injection position was *x* = +0.5 and *y* = −1.6 from bregma coordinates. After skull perforation over the targeted area, the pipette was positioned at *z* = 0.5, and viral particles were slowly injected. After 15 days, mice were sacrificed and perfused with 2% formaldehyde solution. 60 *μ*m thick coronal slices were obtained using a vibratome (Leica). Immunofluorescence and TUNEL assay were performed as described above.

## 3. Results

### 3.1. MCU Overexpression Enhances Mitochondrial Ca^2+^ Uptake in Primary Cortical Neurons

**Figure 1:**
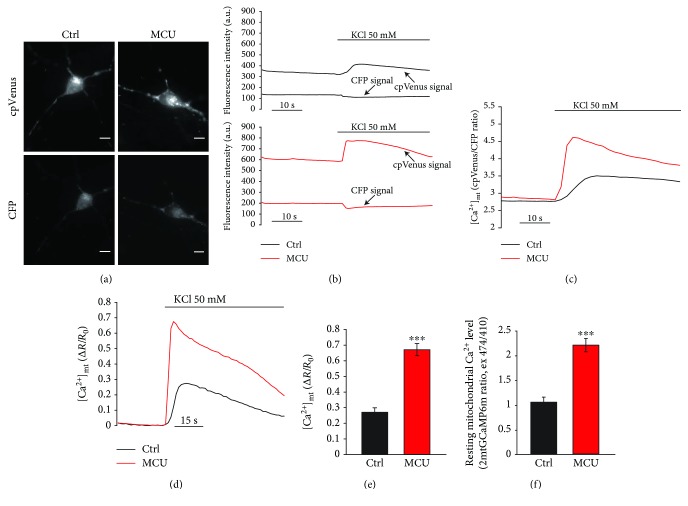
MCU overexpression enhances mitochondrial Ca^2+^ uptake. (a) Representative images of cpVenus and CFP fluorescence in mouse primary cortical neurons cotransfected for 24 hours with 4mtD1cpV probe and either pcDNA3.1 as control (Ctrl) or pcDNA3.1-MCU-flag (MCU). The scale bars represent 10 *μ*m. (b) cpVenus (thick line) and CFP (thin line) fluorescence intensity in neurons transfected as in (a), after KCl depolarization (50 mM). (c) Representative cpVenus/CFP ratio traces of neurons transfected as in (a), after KCl depolarization (50 mM). (d) Representative Δ*R*/*R*_0_ ratio traces and (e) relative Δ*R*/*R*_0_ ratio quantifications of neurons transfected as in (a), after KCl depolarization (50 mM). (f) Resting mitochondrial Ca^2+^ level of mouse primary cortical neurons cotransfected for 24 hours with 2mtGCaMP6m probe and either empty vector mCherry as control (Ctrl) or MCU-mCherry (MCU). Each measurement was performed in at least 30 neurons from 6 different preparations. ^∗∗∗^*p* < 0.0001 compared to control. Detailed statistics are described in Table 1.

In order to dissect the role of MCU in neuronal cells, we used mouse primary cortical neurons cultured from newborn (P_0_-P_2_) C57Bl/6J mice. As previously reported, the overexpression of mitochondrial Ca^2+^ uniporter (MCU) in HeLa cells induces a huge enhancement of mitochondrial Ca^2+^ transients [2]. Based on this observation, we first monitored the mitochondrial Ca^2+^ dynamics in our experimental model. Taking advantages of the FRET-based cameleon-like probe, specifically targeted to mitochondria, 4mtD1cpV [13] (Figure 1(a)), we stimulated both control and MCU-overexpressing cortical neurons with a high [K^+^] causing depolarization-induced Ca^2+^ influx through voltage-sensitive Ca^2+^ channels. Upon stimulation, there was a sharp increase in cpVenus fluorescence and a decrease in CFP fluorescence intensity in both control and MCU-overexpressing primary cortical neurons due to the increased FRET efficiency (Figure 1(b)), resulting in a raise of both cpVenus/CFP ratio and Δ*R*/*R*_0_. As expected, MCU-overexpressing neurons showed a greater increase in the cpVenus/CFP ratio than in control neurons, clearly indicating the enhancement of mitochondrial Ca^2+^ uptake (Figures 1(c), 1(d), and 1(e)). In addition to agonist-induced Ca^2+^ transients, MCU-overexpressing neurons also showed increased resting mitochondrial Ca^2+^ level, as demonstrated by the analysis of the high affinity GFP-based fluorescent Ca^2+^ indicator, 2mtGCamp6m [15, 20, 21]. Indeed, MCU overexpression induces a higher resting mitochondrial [Ca^2+^] compared to mock-transfected neurons (Figure 1(f)).

### 3.2. MCU Overexpression Induces Mitochondrial Fragmentation

**Figure 2:**
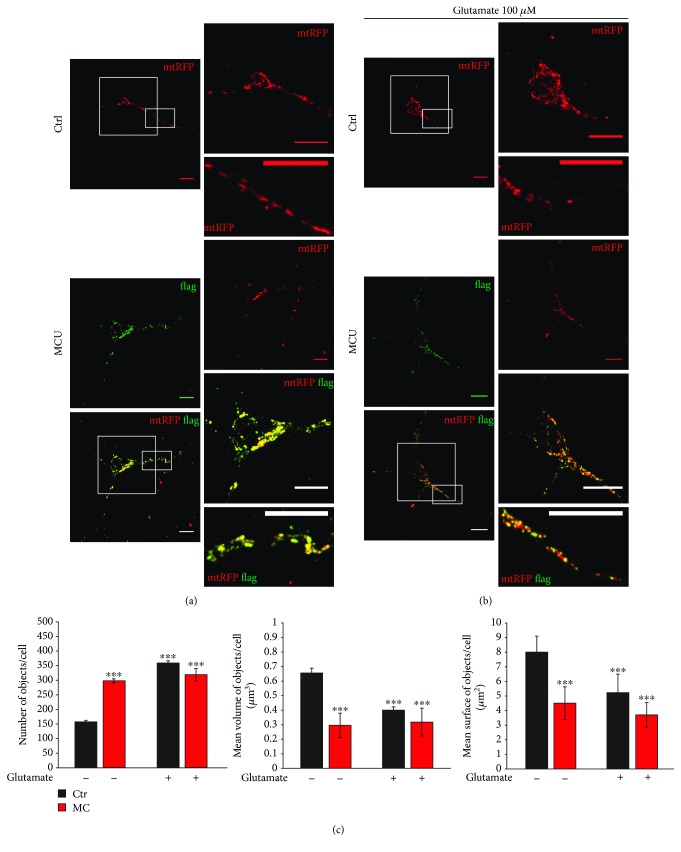
MCU overexpression induces mitochondrial fragmentation. Mouse primary cortical neurons were cotransfected for 24 hours with mtRFP (red) and either empty vector pcDNA3.1 as control (Ctrl) or pcDNA3.1-MCU-flag (MCU), and immunofluorescence was performed as detailed in Methods in absence (a) or in presence (b) of glutamate (100 *μ*M, 1 hour). The scale bars represent 10 *μ*m. (c) Relative quantification of the number of objects per cell (left panel), mean volume of objects per cell (middle panel), and mean surface of objects per cell (right panel) of cells immunostained as in (a) and (b). At least 30 cells from 3 different preparations were analyzed for each condition. ^∗∗∗^*p* < 0.0001 compared to control. Detailed statistics are described in Table 1.

In order to better characterize the consequences of MCU overexpression on mitochondrial function, we investigated the effect of Ca^2+^ on mitochondrial network distribution. To analyze mitochondrial morphology in mouse primary cortical neurons, we used a red fluorescent protein specifically targeted to mitochondria (mtRFP). Confocal microscopy analysis of RFP fluorescence revealed a consistent difference between control and MCU-overexpressing neurons. In mock-transfected neurons, mitochondria appeared elongated and well distributed in the whole cell, in the soma, dendrites, and axons. Conversely, MCU overexpression induced a clear alteration of the overall organelle morphology, with numerous rod-like and fragmented mitochondria, mostly absent at the level of dendrites. Increase in the number of objects per cell was detected, as well as a decrease in their volume and surface (Figures 2(a) and 2(c)). Challenging control neurons with a high concentration of glutamate mimicked this fragmentation (Figures 2(b) and 2(c)). In MCU-overexpressing neurons, glutamate caused no additional impairment of the mitochondrial network.

### 3.3. MCU Overexpression Reduces Neurons Survival *In Vitro*

**Figure 3:**
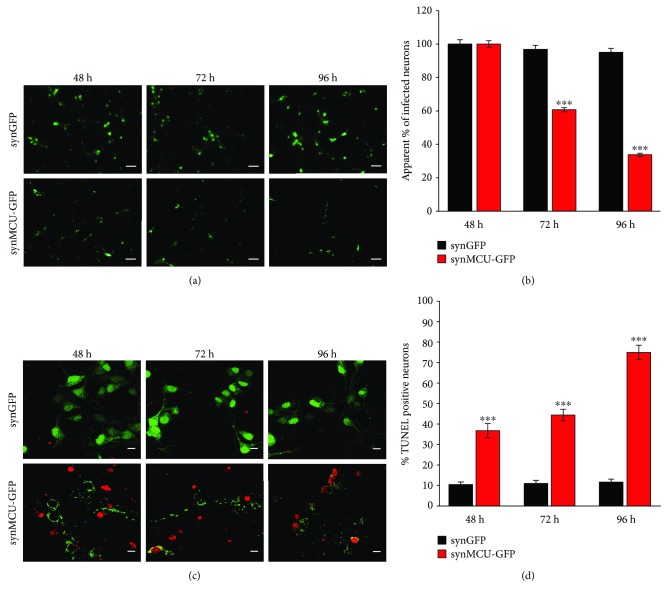
MCU overexpression impairs neuron survival. (a) Mouse primary cortical neurons were infected with synapsin-driven EGFP (synGFP) or MCU-EGFP (synMCU-GFP) adenoviral particles, and representative images were acquired 48, 72, or 96 hours after infection, respectively. The scale bars represent 60 *μ*m. (b) Cells were treated as in (a) and the apparent percentage of GFP-positive neurons relative to the first time point (48 hours) was determined. (c, d) Mouse primary cortical neurons were infected with synapsin-driven EGFP (synGFP) or MCU-EGFP (synMCU-GFP) adenoviral particles. After 48, 72, or 96 hours, cells were fixed and TUNEL assay was performed. (d) Representative images of GFP (green)- and TUNEL (red)-positive neurons. The scale bars represent 10 *μ*m. (d) Quantification of TUNEL-positive neurons for the indicated conditions. At least 60 random fields from 3 different preparations were analyzed. ^∗∗∗^*p* < 0.0001 compared to control. Detailed statistics are described in Table 1.

To better understand the functional consequences of MCU expression in neurons, mouse primary cortical neurons were infected with adenoviruses expressing a synapsin promoter-driven either enhanced green fluorescent protein (synGFP) or a MCU-EGFP (synMCU-GFP) constructs. After infection, the percentage of GFP-positive cells at three different time points (48, 72, and 96 hours after infection) was determined. Neurons infected with the synGFP construct did not show any loss over this period. In contrast, neurons infected with the synMCU-GFP adenoviral particles were progressively lost over time (Figures 3(a) and 3(b)). This suggests that MCU overexpression triggered neuronal death.

To further confirm our findings, the TUNEL assay was employed. Mouse primary cortical neurons were infected as in the previous experiments, and apoptotic cells were labeled with a specific fluorescent marker of fragmented DNA. The percentage of TUNEL-positive cells in synGFP-infected neurons was low and constant over time. Conversely, the percentage of dead neurons progressively increased over time in MCU-overexpressing cultures (Figures 3(c) and 3(d)).

### 3.4. MCU Overexpression Increases Neuronal Death Induced by Glutamate

**Figure 4:**
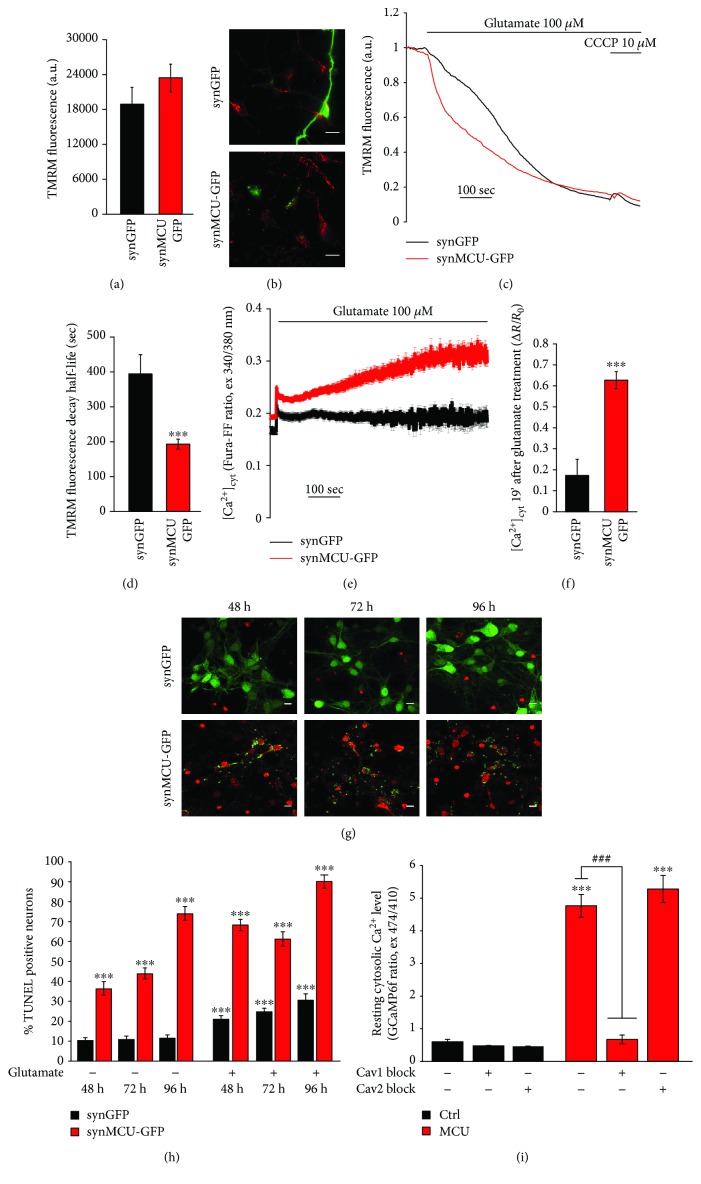
MCU overexpression accelerates neuronal cell death after excitotoxic insults. Mouse primary cortical neurons were infected with synapsin-driven EGFP (synGFP) or MCU-EGFP (synMCU-GFP) adenoviral particles. After 48 hours, cells were loaded with TMRM (20 nM, 30 minutes). (a) TMRM fluorescence intensity in unstimulated resting conditions. (b) Representative images of TMRM staining. The scale bars represent 10 *μ*m. (c) Representative traces of relative TMRM fluorescence intensity. Glutamate (100 *μ*M) and CCCP (10 *μ*M) were added when indicated. (d) Half-life of TMRM fluorescence decay after glutamate treatment. (e, f) Mouse primary cortical neurons were infected with synapsin-driven pEGFP (synGFP) or MCU-GFP (synMCU-GFP) adenoviral particles. After 48 hours, cells were loaded with Fura-FF-AM (5 *μ*M, 30 minutes). (e) Representative traces of Fura-FF fluorescence ratio (excitation at 340 and 380 nm). Glutamate (100 *μ*M) was added when indicated. (f) Average Δ*R*/*R*_0_ values recorded 19 minutes after glutamate treatment. For each condition, at least 30 cells from 3 different preparations were analyzed. (g, h) Mouse primary cortical neurons were infected with synapsin-driven EGFP (synGFP) or MCU-EGFP (synMCU-GFP) adenoviral particles. After 48, 72, and 96 hours, cells were treated with glutamate (100 *μ*M, 1 hour) and then fixed and stained by TUNEL assay. (g) Representative images of GFP (green)- and TUNEL (red)-positive neurons. The scale bars represent 10 *μ*m. (h) Quantification of TUNEL-positive neurons for the indicated conditions. At least 60 random fields from 3 different preparations were analyzed. (i) Resting cytosolic Ca^2+^ level of mouse primary cortical neurons cotransfected for 24 hours with GCaMP6f probe and either empty vector mCherry as control (Ctrl) or MCU-mCherry (MCU), in absence or in presence of Ca_v_1 (nimodipine 5 *μ*M) or Ca_v_2 inhibitors (GVIA 2 *μ*M and M7C 1 *μ*M for Ca_v_2.2 and Ca_v_2.1, respectively). Each measurement was performed in at least 100 neurons from 3 different preparations. ^∗∗∗^*p* < 0.0001 compared to control. Detailed statistics are described in Table 1.

Mitochondrial Ca^2+^ overload can induce opening of the mitochondrial permeability transition pore, leading to loss of inner mitochondrial membrane potential [22]. To determine if MCU-mediated Ca^2+^ loading affected mitochondrial membrane potential, the electrochemical gradient across the inner mitochondrial membrane was monitored using the potentiometric dye tetramethylrhodamine methyl ester (TMRM). In unstimulated resting conditions, MCU overexpression does not cause any evident impairment of TMRM loading, indicating that the membrane potential is intact in this condition (Figures 4(a) and 4(b)). Then, we measured mitochondrial membrane potential changes triggered by glutamate exposure, which should induce MCU-dependent organelle Ca^2+^ overload. As expected, in both control and MCU-overexpressing neurons, there was a loss of mitochondrial membrane potential over time after toxic glutamate exposure. Importantly, glutamate-induced mitochondrial depolarization was twice as fast in MCU-overexpressing neurons (Figures 4(c) and 4(d)).

To assess the contribution of global Ca^2+^ homeostasis during glutamate-induced toxicity, we monitored cytosolic Ca^2+^ dynamics with the low-affinity Ca^2+^-sensitive ratiometric probe Fura-FF. In control neurons, glutamate induced an initial fast increase in [Ca^2+^], followed by a sustained plateau phase that lasts for several minutes. Conversely, in MCU-overexpressing neurons, glutamate-induced [Ca^2+^] elevations progressively rise over time (Figures 4(e) and 4(f)). Accordingly, treatment with a toxic dose of glutamate increased the percentage of apoptotic cells in MCU-overexpressing neurons (Figures 4(g) and 4(h)). These experiments are consistent with the indication that MCU overexpression sensitizes neurons to excitotoxic stimuli leading to cell death.

Surprisingly, we detected a slight evelation in resting cytosolic [Ca^2+^] in MCU-overexpressing neurons (Figure 4(e)). To better address this point, we used the high-affinity GFP-based fluorescent Ca^2+^ indicator, GCamp6f [16]. This probe efficiently revealed a significant increase of basal cytosolic Ca^2+^ level in MCU-overexpressing neurons (Figure 4(i)), suggesting an impairment in the Ca^2+^ permeability at the plasma membrane. We thus wondered whether this phenotype could be rescued by inhibiting either Ca_v_1 or Ca_v_2 channels. As shown in Figure 4(i), Ca_v_2 inhibition had no consequences on resting cytosolic [Ca^2+^] in both control and MCU-overexpressing neurons. Conversely, pharmacological block of Ca_v_1 channels in MCU-overexpressing cells efficiently decreased resting cytosolic [Ca^2+^] to values similar to control neurons.

### 3.5. MCU Overexpression *In Vivo* Induces Brain Tissue Degeneration

**Figure 5:**
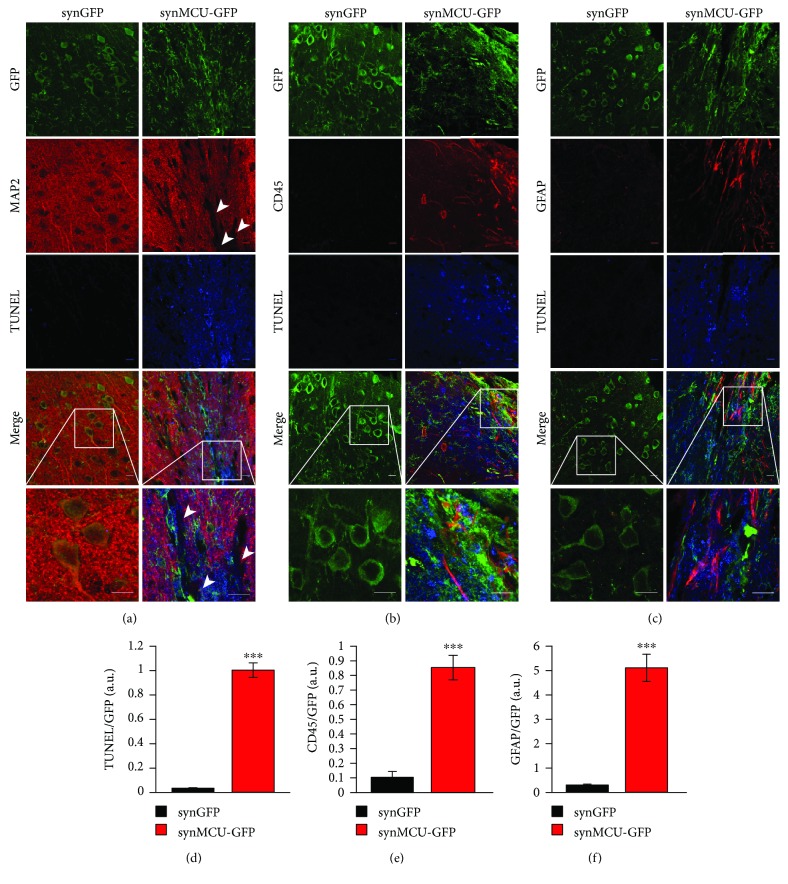
MCU-induced cortical degeneration. Synapsin promoter-driven EGFP (synGFP) or MCU-EGFP (synMCU-GFP) adenoviral particles were stereotaxically injected in the cortex of C57Bl/6J mice. After 15 days, mice were sacrificed and immunohistochemistry of coronal brain slices was performed. GFP immunofluorescence is shown in green. MAP2 (a), CD45 (b), and GFAP (c) are shown in red. Dead cells were detected through TUNEL assay (in blue). The scale bars represent 10 *μ*m. Quntification of TUNEL (d), CD45 (e), and GFAP (f) fluorescence intensity above threshold on GFP fluorescence intensity. Images are representative of 3 different mice, derived from 3 independent infections. ^∗∗∗^*p* < 0.0001 compared to control. Detailed statistics are described in Table 1.

Our *in vitro* experiments clearly indicate that MCU overexpression causes mitochondrial Ca^2+^ overload and triggers neuronal death. We finally wondered whether this happens also *in vivo*. We thus injected control and MCU-encoding adenoviral particles (under the control of the neuron-specific synapsin promoter) into the cortex of C57Bl/6J mice, using the stereotaxical approach [19]. After 15 days, we verified the transgene expression in coronal brain slices of injected mice (Figure 5). In parallel, we performed immunostaining of the neuron-specific cytoskeletal protein MAP2 (microtubule-associated protein 2) on the same sections. In GFP-injected brains, the vast majority of the infected neurons were also positively labeled with MAP2 antibody. On the contrary, in MCU-injected brains, many infected neurons displayed decreased MAP2 staining (Figure 5(a)), suggesting ongoing neuron degeneration [23]. Accordingly, TUNEL staining showed extensive cell death in MCU-injected but not in GFP-injected brains (Figures 5(a)‑5(d)).

In neurodegenerative disorders, neuronal loss is accompanied by the appearance of extracellular debris, elevated levels of proinflammatory cytokines, and activated microglia [24–27]. In the cortex of GFP-injected mice, little microglial activation could be detected through CD45 immunoreactivity. Conversely, in the cortex of MCU-overexpressing mice, a robust CD45 staining was evident (Figures 5(b) and 5(e)).

Also, astrocytes play a central role in the maintenance of CNS functions, and hypertrophic astrocytes always follow brain injury [28–30]. Glial fibrillary acidic protein (GFAP) can thus be used as a reliable marker of reactive astrocytes. Only few GFAP-positive cells were observed in GFP-injected mice. However, a clear accumulation of GFAP immunoreactive astrocytes was observed in MCU-injected mice (Figures 5(c) and 5(f)). Thus, *in vivo*, neuronal MCU overexpression triggered signs of neuronal degeneration, including microglial activation, gliosis, and increased TUNEL staining.

## 4. Discussion

The recent elucidation of MCU complex components has allowed the interpretation of the physiopathological role of mitochondrial Ca^2+^ homeostasis through genetic approaches, overcoming the paucity of specific pharmacological tools. Several lines of evidence show that mitochondrial Ca^2+^ is a pleiotropic signal. On the one hand, elevation of Ca^2+^ in the organelle matrix can efficiently boost ATP production through a concerted positive regulation on both the rate-limiting enzymes of the TCA cycle and the oxidative phosphorylation complexes. On the other hand, excessive Ca^2+^ accumulation is the best characterized trigger of PTP opening that leads to cell death [5].

However, this notion has been critically challenged by the first attempt to knockout the MCU gene in mammals. Surprisingly, mice completely lacking MCU show no overt phenotype [31]. Nonetheless, it must be stressed that this result has been obtained in a mixed genetic background [32], while MCU ablation in the pure C57Bl/6 strain leads to complete embryonic lethality [32–34], as one would expect. Similarly, constitutive knockout of EMRE, an essential MCU modulator, also leads to complete preweaning lethality [35]. Even mice with genetic ablation of MICU1, a key modulator of MCU opening [21, 36–38], die within hours of birth [39, 40]. In addition, human patients with loss of function mutations in MICU1 display a clinical phenotype with multiple defects that recapitulates some clinical features of mitochondrial diseases [20, 41].

All these observations highlight the importance of mitochondrial Ca^2+^ uptake and suggest that MCU inhibition during development can trigger confounding adaptive mechanisms that potentially undervalue the physiopathological role of MCU. We thus reasoned that the modulation of MCU levels after development is a valuable strategy to understand the role of mitochondrial Ca^2+^ signaling in health and disease. Until now, this approach has been used *in vivo* only for the striated muscles, both cardiac [33, 42] and skeletal [43]. Here, we show that, in primary cortical neurons, overexpression of MCU leads to increased mitochondrial Ca^2+^ levels in both unstimulated cells (Figure 1(f)) and after plasma membrane depolarization (Figures 1(a)‑1(e)), as already reported [10]. However, in these cells, MCU overexpression is clearly toxic and leads to cell death *per se* (Figure 3), even in the absence of additional external stimuli. Interestingly, this effect is rarely seen in other cell types [43, 44], thus suggesting that neurons are particularly susceptible to mitochondrial Ca^2+^-mediated cell death. In line with this, MCU overexpression causes fragmentation of mitochondrial network (Figure 2) and increased basal cytosolic [Ca^2+^] (Figure 4(i)), i.e., two early markers of neuronal dysfunction. Whether or not these two phenomena depend one on the other in this experimental context is still unclear. However, recent data suggest that this could be the case. Indeed, elevations in [Ca^2+^] trigger a process named mitochondrial shape transition (MiST), leading to organelle fragmentation even in the absence of PTP opening and ΔΨ_m_ loss [45]. Therefore, organelle fragmentation in normal resting condition can be secondary to the chronic increase of basal [Ca^2+^]_cyt_. MCU overexpression mimics the effects of the excitotoxic stimulus treatment, precisely glutamate, on both mitochondrial morphology and neuronal cell death (Figures 2(b), 2(c) and 4(g)–4(h)). Furthermore, MCU-overexpressing neurons show early loss of mitochondrial membrane potential (Figures 4(b)‑4(d)) and cellular Ca^2+^ overload (Figures 4(e)‑4(f)). However, the exact mechanism linking organelle Ca^2+^ overload to cell death still needs to be elucidated. Indeed, several potential mechanisms could account for this effect, including mitochondrial fragmentation, cytosolic calcium overload, Ca^2+^-dependent disruption of Miro1/KIF5B/tubulin complex, PTP opening, ROS production, and calpain activation. Similarly, we did not analyze which specific type of death is occurring in our experimental setup. The exact mechanism and the relative contribution of each of these pathways will require additional and dedicated efforts.

Overall, our *in vitro* data unambiguously support the idea that exaggerated mitochondrial Ca^2+^ uptake in neurons is primarily a cell death trigger. We thus moved to an *in vivo* system by delivering adenoviral particles encoding for a synapsin promoter-driven MCU construct within the mouse cortex. In this scenario, MCU overexpression clearly causes neuronal cell death, as demonstrated by the impairment of cytoskeletal organization and the appearance of TUNEL-positive nuclei (Figures 5(a) and 5(d)). In addition, activation of both microglia (Figures 5(b) and 5(e)) and astrocytes (Figures 5(c) and 5(f)) was evident in MCU-overexpressing brains, thus recapitulating the main features of neurodegeneration. This demonstrates that mitochondrial Ca^2+^ overload triggers *per se* neuronal cell death also *in vivo*. In line with this view, postnatal ablation of MCU in neurons has been recently shown to protect from ischemia/reperfusion injury in the brain [46].

Intriguingly, MCU overexpression alone is not sufficient to cause degeneration in other tissues. For instance, in skeletal muscle increased mitochondrial Ca^2+^ uptake stimulates protein synthesis, activates hypertrophic pathways, and increases the overall muscle mass [43]. Conversely, ablation of MICU1, i.e., a genetic condition that increases resting mitochondrial [Ca^2+^] and thus predisposes to organelle Ca^2+^ overload, causes no gross alterations in the healthy liver but potently prevents tissue regeneration upon stress conditions (e.g., partial hepatectomy). These differences can however be explained by considering that mitochondrial Ca^2+^ is a pleiotropic signal that can be differentially decoded according to the global characteristics of cell-specific signaling toolkit. On the one hand, Ca^2+^-dependent stimulation of oxidative metabolism appears to be critical for cell survival [47]. On the other hand, excessive Ca^2+^ accumulation inside organelle matrix is the key determinant of permeability transition and cell death. It is widely believed that neuronal functions (e.g., action potentials, exocytosis) are highly energy-demanding. However, metabolic flexibility (i.e., the capacity to adapt fuel oxidation to fuel availability) is paradoxically rather limited in neurons. They strongly rely on glucose or astrocyte-derived lactate to sustain ATP production, whereas oxidation of fatty acid plays a marginal role [48, 49]. Conversely, most of the other tissues, including those with high energy demand such as the skeletal muscle or heart, can easily utilize both carbohydrates and lipids for their oxidative metabolism. It must be noted that in our experimental conditions, MCU is overexpressed without the concomitant overexpression of its endogenous regulators (i.e., MICU1 and/or MICU2). This leads to increased resting intramitochondrial [Ca^2+^] (Figure 1(f)), thus indicating that a futile Ca^2+^ cycling across the IMM is occurring in these cells. As a consequence, the increased flux through the TCA cycle determined by elevated mitochondrial [Ca^2+^] is likely to be undermined by the energy waste caused by this futile cycle. A similar scenario has been recently proposed in cells derived from patients lacking MICU1, i.e., another condition that chronically increases matrix [Ca^2+^] [50]. Tissues with higher metabolic flexibility such as skeletal muscle or liver can likely better compensate this energy drain in normal conditions. Conversely, MCU overexpression in neurons appears to be highly toxic also in the absence of any stress signal. In this cellular model, the impairment of MCU complex leads to mitochondrial Ca^2+^ overload with consequent cell death [51].

Overall, these data clearly indicate that exagerated organelle Ca^2+^ levels can be a key mechanism underlying neurodegeneration, thus uncovering a new putative target with enormous therapeutic potential.

## 5. Conclusions

In conclusion, we demonstrated that mitochondrial Ca^2+^ overload is *per se* sufficient to cause neuronal cell death both *in vitro* and *in vivo*, thus highlighting a potential key step in neurodegeneration. This evidence opens the possibility to clinical intervention through the regulation of intracellular Ca^2+^ signaling and, in particular, through the modulation of mitochondrial Ca^2+^ uptake.

## Figures and Tables

**Table 1:** Descriptive statistics.

Experiment	Measurements	Mean ± S.E.	No. of samples	No. of experiments	Figure
Ctrl	[Ca^2+^]_mt_ (Δ*R*/*R*_0_)	0.26 ± 0.03	30	6	1(e)
MCU	[Ca^2+^]_mt_ (Δ*R*/*R*_0_)	0.67 ± 0.04	30	6	1(e)
Ctrl	Resting mitochondrial Ca^2+^ level (474/410 ratio)	1.04 ± 0.10	30	6	1(f)
MCU	Resting mitochondrial Ca^2+^ level (474/410 ratio)	2.19 ± 0.13	30	6	1(f)
Ctrl	Number of objects/cell	158 ± 5	30	3	2(c)
MCU	Number of objects/cell	299 ± 7	30	3	2(c)
Ctrl+glutamate	Number of objects/cell	360 ± 8	30	3	2(c)
MCU+glutamate	Number of objects/cell	320 ± 21	30	3	2(c)
Ctrl	Mean volume of objects/cell (*μ*m^3^)	0.66 ± 0.03	30	3	2(c)
MCU	Mean volume of objects/cell (*μ*m^3^)	0.30 ± 0.08	30	3	2(c)
Ctrl+glutamate	Mean volume of objects/cell (*μ*m^3^)	0.40 ± 0.02	30	3	2(c)
MCU+glutamate	Mean volume of objects/cell (*μ*m^3^)	0.32 ± 0.10	30	3	2(c)
Ctrl	Mean surface of objects/cell (*μ*m^2^)	8.0 ± 1.1	30	3	2(c)
MCU	Mean surface of objects/cell (*μ*m^2^)	4.5 ± 1.1	30	3	2(c)
Ctrl+glutamate	Mean surface of objects/cell (*μ*m^2^)	5.2 ± 1.3	30	3	2(c)
MCU+glutamate	Mean surface of objects/cell (*μ*m^2^)	3.7 ± 0.8	30	3	2(c)
synGFP_48h	Apparent-infected neurons (%)	100.0 ± 2.6	60	3	3(b)
synMCU-GFP_48h	Apparent-infected neurons (%)	100.0 ± 2.0	60	3	3(b)
synGFP_72h	Apparent-infected neurons (%)	96.8 ± 2.5	60	3	3(b)
synMCU-GFP_72h	Apparent-infected neurons (%)	60.7 ± 1.3	60	3	3(b)
synGFP_96h	Apparent-infected neurons (%)	95.1 ± 2.4	60	3	3(b)
synMCU-GFP_96h	Apparent-infected neurons (%)	33.8 ± 0.8	60	3	3(b)
synGFP_48h	TUNEL-positive neurons (%)	10.6 ± 1.3	60	3	3(d)
synMCU-GFP_48h	TUNEL-positive neurons (%)	37.3 ± 3.5	60	3	3(d)
synGFP_72h	TUNEL-positive neurons (%)	11.2 ± 1.6	60	3	3(d)
synMCU-GFP_72h	TUNEL-positive neurons (%)	45.0 ± 2.8	60	3	3(d)
synGFP_96h	TUNEL-positive neurons (%)	11.8 ± 1.4	60	3	3(d)
synMCU-GFP_96h	TUNEL-positive neurons (%)	75.9 ± 3.6	60	3	3(d)
synGFP	TMRM fluorescence (a.u.)	19310 ± 2989	10	3	4(a)
synMCU-GFP	TMRM fluorescence (a.u.)	23974 ± 2439	10	3	4(a)
synGFP	TMRM fluorescence decay half-life (sec)	394.2 ± 55.3	10	3	4(d)
synMCU-GFP	TMRM fluorescence decay half-life (sec)	194.4 ± 14.3	10	3	4(d)
synGFP	[Ca^2+^]_cyt_ 19′ after glutamate treatment (Δ*R*/*R*_0_)	0.17 ± 0.08	30	3	4(f)
synMCU-GFP	[Ca^2+^]_cyt_ 19′ after glutamate treatment (Δ*R*/*R*_0_)	0.63 ± 0.04	30	3	4(f)
synGFP_48h (+GLUT)	TUNEL-positive neurons (%)	21.0 ± 1.8	60	3	4(h)
synMCU-GFP_48h (+GLUT)	TUNEL-positive neurons (%)	68.3 ± 2.8	60	3	4(h)
synGFP_72h (+GLUT)	TUNEL-positive neurons (%)	24.7 ± 1.8	60	3	4(h)
synMCU-GFP_72h (+GLUT)	TUNEL-positive neurons (%)	61.2 ± 3.6	60	3	4(h)
synGFP_96h (+GLUT)	TUNEL-positive neurons (%)	30.5 ± 3.3	60	3	4(h)
synMCU-GFP_96h (+GLUT)	TUNEL-positive neurons (%)	90.1 ± 3.3	60	3	4(h)
Ctrl	Resting cytosolic Ca^2+^ level (474/410 ratio)	0.60 ± 0.07	100	3	4(i)
Ctrl+Cav1 block	Resting cytosolic Ca^2+^ level (474/410 ratio)	0.48 ± 0.01	100	3	4(i)
Ctrl+Cav2 block	Resting cytosolic Ca^2+^ level (474/410 ratio)	0.46 ± 0.01	100	3	4(i)
MCU	Resting cytosolic Ca^2+^ level (474/410 ratio)	4.77 ± 0.35	100	3	4(i)
MCU+Cav1 block	Resting cytosolic Ca^2+^ level (474/410 ratio)	0.67 ± 0.13	100	3	4(i)
MCU+Cav2 block	Resting cytosolic Ca^2+^ level (474/410 ratio)	5.28 ± 0.41	100	3	4(i)
synGFP	TUNEL/GFP (a.u.)	0.032 ± 0.005	12	3	5(d)
synMCU-GFP	TUNEL/GFP (a.u.)	1.001 ± 0.060	12	3	5(d)
synGFP	CD45/GFP (a.u.)	0.104 ± 0.040	10	3	5(e)
synMCU-GFP	CD45/GFP (a.u.)	0.854 ± 0.084	13	3	5(e)
synGFP	GFAP/GFP (a.u.)	0.298 ± 0.040	8	3	5(f)
synMCU-GFP	GFAP/GFP (a.u.)	5.100 ± 0.559	10	3	5(f)

## Data Availability

The main data supporting the findings of this study are listed in Table 1.
